# Bis[tetra­aqua­(1,10-phenanthroline-κ^2^ *N*,*N*′)cobalt(II)] hexa­aqua­cobalt(II) bis­[3,5-bis­(carboxyl­atometh­oxy)benzoate] tetra­hydrate

**DOI:** 10.1107/S1600536810046386

**Published:** 2010-11-17

**Authors:** Hui-Ying Song, Xiao-Juan Wang, Shi-Zhen Qiu, Yun-Long Feng

**Affiliations:** a College of Chemistry and Life Sciences, Zhejiang Normal University, Jinhua, Zhejiang 321004, People’s Republic of China; b Zhejiang Key Laboratory for Reactive Chemistry on Solid Surfaces, Institute of Physical Chemistry, Zhejiang Normal University, Jinhua, Zhejiang 321004, People’s Republic of China

## Abstract

The title compound, [Co(C_12_H_8_N_2_)(H_2_O)_4_]_2_[Co(H_2_O)_6_](C_11_H_7_O_8_)_2_·4H_2_O, was obtanied by the reaction of cobalt acetate with 3,5-bis­(carb­oxy­meth­oxy)benzoic acid and 1,10-phenanthroline. The asymmetric unit contains one tetra­aqua­(1,10-phenanthroline)cobalt(II) cation, one half of a hexa­aqua­cobalt(II) cation that is completed by inversion symmetry, one 3,5-bis­(carboxyl­atometh­oxy)benzoate trianion and two lattice water mol­ecules. The two Co^II^ atoms each show a slightly distorted octa­hedral coordination (CoO_6_ and CoO_4_N_2_). The cations, anions and lattice water mol­ecules are linked by an intricate network of O—H⋯O hydrogen bonds into a three-dimensional structure.

## Related literature

For background to multicarboxyl­ate ligands, see: Cao *et al.* (2002 ▶); Dai *et al.* (2002 ▶); He *et al.* (2008 ▶); Rowsell *et al.* (2005 ▶); Wang *et al.* (2005 ▶).
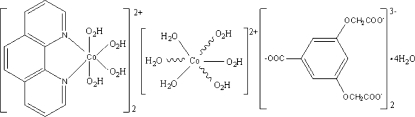


## Experimental

### 

#### Crystal data

[Co(C_12_H_8_N_2_)(H_2_O)_4_]_2_[Co(H_2_O)_6_](C_11_H_7_O_8_)_2_·4H_2_O
*M* *_r_* = 1395.82
Monoclinic, 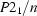

*a* = 7.0924 (1) Å
*b* = 20.3779 (4) Å
*c* = 20.1810 (3) Å
β = 99.063 (1)°
*V* = 2880.31 (8) Å^3^
*Z* = 2
Mo *K*α radiation
μ = 0.96 mm^−1^
*T* = 296 K
0.22 × 0.15 × 0.07 mm

#### Data collection

Bruker APEXII area-detector diffractometer
Absorption correction: multi-scan (*SADABS*; Sheldrick, 1996 ▶) *T*_min_ = 0.839, *T*_max_ = 0.933
21923 measured reflections
5080 independent reflections
3751 reflections with *I* > 2σ(*I*)
*R*_int_ = 0.054

#### Refinement

*R*[*F* ^2^ > 2σ(*F* ^2^)] = 0.039
*wR*(*F* ^2^) = 0.100
*S* = 1.09
5080 reflections
450 parameters
27 restraints
H atoms treated by a mixture of independent and constrained refinement
Δρ_max_ = 0.31 e Å^−3^
Δρ_min_ = −0.30 e Å^−3^

### 

Data collection: *APEX2* (Bruker, 2006 ▶); cell refinement: *SAINT* (Bruker, 2006 ▶); data reduction: *SAINT*; program(s) used to solve structure: *SHELXS97* (Sheldrick, 2008 ▶); program(s) used to refine structure: *SHELXL97* (Sheldrick, 2008 ▶); molecular graphics: *SHELXTL* (Sheldrick, 2008 ▶); software used to prepare material for publication: *SHELXTL*.

## Supplementary Material

Crystal structure: contains datablocks I, global. DOI: 10.1107/S1600536810046386/wm2426sup1.cif

Structure factors: contains datablocks I. DOI: 10.1107/S1600536810046386/wm2426Isup2.hkl

Additional supplementary materials:  crystallographic information; 3D view; checkCIF report

## Figures and Tables

**Table 1:** Selected bond lengths (Å)

Co1—O5*W*	2.0144 (19)
Co1—O6*W*	2.112 (2)
Co1—O7*W*	2.115 (3)
Co2—O2*W*	2.072 (2)
Co2—O1*W*	2.093 (2)
Co2—O4*W*	2.102 (2)
Co2—N1	2.109 (2)
Co2—O3*W*	2.118 (2)
Co2—N2	2.157 (2)

**Table 2:** Hydrogen-bond geometry (Å, °)

*D*—H⋯*A*	*D*—H	H⋯*A*	*D*⋯*A*	*D*—H⋯*A*
O4*W*—H4*WA*⋯O7^i^	0.84 (2)	1.88 (2)	2.713 (3)	173 (3)
O2*W*—H2*WA*⋯O8^i^	0.86 (2)	1.86 (2)	2.690 (3)	163 (3)
O8*W*—H8*WA*⋯O1^i^	0.84 (2)	1.91 (2)	2.732 (3)	163 (4)
O4*W*—H4*WB*⋯O2^ii^	0.84 (2)	1.91 (2)	2.731 (3)	169 (3)
O7*W*—H7*WA*⋯O9*W*^ii^	0.84 (2)	2.01 (2)	2.818 (3)	164 (3)
O8*W*—H8*WB*⋯O4^ii^	0.85 (2)	2.29 (3)	2.945 (3)	134 (4)
O3*W*—H3*WA*⋯O7^iii^	0.83 (2)	2.12 (2)	2.881 (3)	152 (3)
O5*W*—H5*WA*⋯O1^iii^	0.84 (2)	1.77 (2)	2.609 (3)	175 (4)
O1*W*—H1*WB*⋯O7^iii^	0.84 (2)	1.88 (2)	2.711 (3)	170 (3)
O6*W*—H6*WB*⋯O5^iv^	0.81 (2)	2.00 (2)	2.812 (3)	177 (3)
O5*W*—H5*WB*⋯O4^iv^	0.83 (2)	1.89 (2)	2.715 (3)	174 (4)
O3*W*—H3*WB*⋯O4^iv^	0.79 (2)	2.25 (2)	3.029 (3)	168 (4)
O3*W*—H3*WB*⋯O3^iv^	0.79 (2)	2.54 (3)	3.080 (3)	127 (3)
O9*W*—H9*WB*⋯O2^v^	0.82 (2)	2.00 (2)	2.818 (3)	172 (4)
O7*W*—H7*WB*⋯O9*W*^vi^	0.83 (2)	1.97 (2)	2.805 (4)	174 (4)
O1*W*—H1*WA*⋯O5^vii^	0.82 (2)	1.97 (2)	2.785 (3)	172 (4)
O6*W*—H6*WA*⋯O2	0.79 (2)	2.00 (2)	2.775 (3)	169 (3)
O2*W*—H2*WB*⋯O8*W*	0.85 (2)	1.88 (2)	2.723 (3)	177 (3)
O9*W*—H9*WA*⋯O5	0.83 (2)	2.00 (2)	2.831 (3)	173 (3)

Symmetry codes: (i) 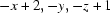; (ii) 
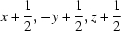
; (iii) 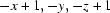; (iv) 
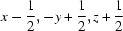
; (v) 
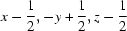
; (vi) 
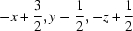
; (vii) 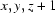.

**Fig. 1.**
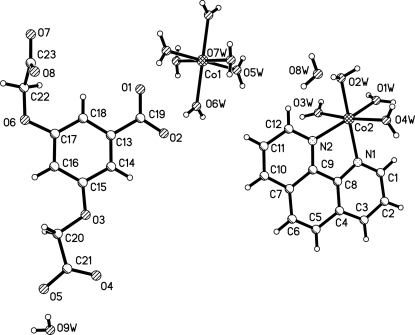
Perspective view of the molecular entities of (I), showing the atom-numbering scheme. Displacement ellipsoids are drawn at the 30% probability level.

**Fig. 2.**
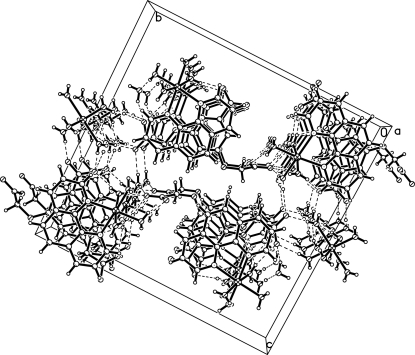
The three-dimensional set up of structure of (I), viewed down [100]. Dashed lines indicate hydrogen bonds.

## supplementary crystallographic information

### Comment

Multibenzenecarboxylate ligands, such as terephthalic acid, 1,3,5-benzenetricarboxylic acid, or 1,2,4,5-benzenetetracarboxylic acid, have been employed in the construction of numerous framework compounds (Dai *et al.*, 2002; Rowsell *et al.*, 2005; Wang *et al.*, 2005; Cao *et al.*, 2002). Herein, on the basis of the rigidity of 3,5-dihydroxybenzoic acid, we successfully designed a new multicarboxylate ligand, *viz*. 3,5-bis-carboxymethoxy-benzoic acid (C_11_H_10_O_8_) (He *et al.*, 2008). In this work, we report the synthesis and structure of a new compound, [Co(C_12_H_8_N_2_)(H_2_O)_4_]_2_[Co(H_2_O)_6_](C_11_H_7_O_8_)_2_^.^4H_2_O, (I).

A perspective view of the molecular entities of compound (I) is presented in Fig.1. The asymmetric unit consists of one [Co(C_12_H_8_N_2_)(H_2_O)_4_]^2+^, half a [Co(H_2_O)_6_]^2+^ cation (1 symmetry), one (C_11_H_7_O_8_)_2_^3-^ anion, and two lattice water molecules. In the cations, the Co^II^ atoms show a slightly distorted octahedral coordination (CoO_6_ and CoO_4_N_2_, respectively). In the anion, one of the carboxymethyl groups is almost co-planar to the benzene ring with the dihedral angle of 3.5 (1)°, while the formate group makes a dihedral angle of 17.2 (1)° with the benzene ring. The other carboxymethyl group is almost perpendicular to the benzene ring with the torsion angle C17—O6—C22—C23 of 81.3 (3)°. Together with lattice water molecules, the carboxylic O atoms act as acceptors of O—H···O hydrogen bonds forming a three-dimensional structure (Fig. 2).

### Experimental

A mixture of 3,5-bis-carboxymethoxy-benzoic acid (0.373 g, 1.50 mmol), Co(CH_3_COO)_2_^.^4H_2_O (0.282 g, 1.00 mmol), 1,10-phenanthroline (0.049 g, 0.25 mmol), and Na_2_CO_3_ (0.079 g, 0.75 mmol) in C_2_H_5_OH (2 ml)/H_2_O (16 ml) was placed in a Teflon-lined stainless steel vessel and heated at 433 K for 72 h, and then cooled to room temperature over 3 days. Then the reaction mixture was filtered and well-shaped pink crystals of compound (I) were obtained from the mother liquor by slow evaporation at room temperature for several days.

### Refinement

The carbon-bound H-atoms were positioned geometrically and included in the refinement using a riding model [aromatic C—H 0.93Å and aliphatic C—H 0.97 Å, *U*_iso_(H) = 1.2*U*_eq_(C)]. The oxygen-bound H-atoms were located in difference Fourier maps and refined with the O—H distance restrained to 0.85 Å and *U*_iso_(H) = 1.2*U*_eq_(O).

### Crystal data

**Table:**

[Co(C_12_H_8_N_2_)(H_2_O)_4_]_2_[Co(H_2_O)_6_](C_11_H_7_O_8_)_2_·4H_2_O	*F*(000) = 1446
*M**_r_* = 1395.82	*D*_x_ = 1.609 Mg m^−^^3^
Monoclinic, *P*2_1_/*n*	Mo *K*α radiation, λ = 0.71073 Å
Hall symbol: -P 2yn	Cell parameters from 3158 reflections
*a* = 7.0924 (1) Å	θ = 1.4–25.0°
*b* = 20.3779 (4) Å	µ = 0.96 mm^−^^1^
*c* = 20.1810 (3) Å	*T* = 296 K
β = 99.063 (1)°	Block, pink
*V* = 2880.31 (8) Å^3^	0.22 × 0.15 × 0.07 mm
*Z* = 2	

### Data collection

**Table:**

Bruker APEXII area-detector diffractometer	5080 independent reflections
Radiation source: fine-focus sealed tube	3751 reflections with *I* > 2σ(*I*)
graphite	*R*_int_ = 0.054
ω scans	θ_max_ = 25.0°, θ_min_ = 1.4°
Absorption correction: multi-scan (*SADABS*; Sheldrick, 1996)	*h* = −7→8
*T*_min_ = 0.839, *T*_max_ = 0.933	*k* = −24→24
21923 measured reflections	*l* = −23→23

### Refinement

**Table:**

Refinement on *F*^2^	Primary atom site location: structure-invariant direct methods
Least-squares matrix: full	Secondary atom site location: difference Fourier map
*R*[*F*^2^ > 2σ(*F*^2^)] = 0.039	Hydrogen site location: inferred from neighbouring sites
*wR*(*F*^2^) = 0.100	H atoms treated by a mixture of independent and constrained refinement
*S* = 1.09	*w* = 1/[σ^2^(*F*_o_^2^) + (0.0472*P*)^2^] where *P* = (*F*_o_^2^ + 2*F*_c_^2^)/3
5080 reflections	(Δ/σ)_max_ = 0.001
450 parameters	Δρ_max_ = 0.31 e Å^−^^3^
27 restraints	Δρ_min_ = −0.30 e Å^−^^3^

### Special details

**Table:**

Geometry. All e.s.d.'s (except the e.s.d. in the dihedral angle between two l.s. planes) are estimated using the full covariance matrix. The cell e.s.d.'s are taken into account individually in the estimation of e.s.d.'s in distances, angles and torsion angles; correlations between e.s.d.'s in cell parameters are only used when they are defined by crystal symmetry. An approximate (isotropic) treatment of cell e.s.d.'s is used for estimating e.s.d.'s involving l.s. planes.
Refinement. Refinement of *F*^2^ against ALL reflections. The weighted *R*-factor *wR* and goodness of fit *S* are based on *F*^2^, conventional *R*-factors *R* are based on *F*, with *F* set to zero for negative *F*^2^. The threshold expression of *F*^2^ > σ(*F*^2^) is used only for calculating *R*-factors(gt) *etc*. and is not relevant to the choice of reflections for refinement. *R*-factors based on *F*^2^ are statistically about twice as large as those based on *F*, and *R*- factors based on ALL data will be even larger.

### Fractional atomic coordinates and isotropic or equivalent isotropic displacement parameters (Å^2^)

**Table:**

	*x*	*y*	*z*	*U*_iso_*/*U*_eq_	
Co1	0.5000	0.0000	0.5000	0.03353 (17)	
Co2	0.74606 (6)	0.243825 (19)	0.763479 (18)	0.03271 (14)	
N1	0.7089 (3)	0.34589 (12)	0.74971 (11)	0.0339 (6)	
N2	0.7521 (3)	0.25550 (12)	0.65761 (11)	0.0344 (6)	
O1W	0.6596 (4)	0.23471 (14)	0.85757 (11)	0.0551 (7)	
H1WA	0.696 (4)	0.2535 (16)	0.8930 (12)	0.066*	
H1WB	0.548 (3)	0.2210 (18)	0.8584 (16)	0.066*	
O1	0.7285 (4)	0.00598 (10)	0.32671 (11)	0.0589 (7)	
O2W	0.8488 (3)	0.14848 (11)	0.76823 (11)	0.0441 (6)	
H2WA	0.921 (4)	0.1411 (17)	0.8057 (9)	0.053*	
H2WB	0.926 (4)	0.1379 (16)	0.7422 (11)	0.053*	
O2	0.6887 (3)	0.11208 (10)	0.34103 (9)	0.0383 (5)	
O3W	0.4615 (3)	0.21473 (13)	0.72790 (10)	0.0486 (6)	
H3WA	0.395 (4)	0.1961 (15)	0.7526 (12)	0.058*	
H3WB	0.413 (4)	0.2101 (17)	0.6903 (8)	0.058*	
O3	0.7342 (3)	0.20327 (10)	0.11420 (9)	0.0472 (6)	
O4	0.7996 (3)	0.32426 (10)	0.08547 (9)	0.0479 (6)	
O4W	1.0173 (3)	0.26858 (11)	0.81544 (12)	0.0474 (6)	
H4WA	1.106 (4)	0.2421 (12)	0.8289 (16)	0.057*	
H4WB	1.072 (4)	0.3051 (9)	0.8180 (16)	0.057*	
O5W	0.4188 (4)	0.04985 (11)	0.57696 (12)	0.0663 (8)	
H5WA	0.367 (5)	0.0307 (15)	0.6061 (15)	0.080*	
H5WB	0.389 (6)	0.0890 (9)	0.5780 (18)	0.080*	
O5	0.7541 (3)	0.30672 (10)	−0.02457 (9)	0.0382 (5)	
O6W	0.4901 (3)	0.08954 (10)	0.44683 (10)	0.0457 (6)	
H6WA	0.533 (4)	0.0971 (15)	0.4139 (12)	0.055*	
H6WB	0.420 (4)	0.1186 (13)	0.4560 (15)	0.055*	
O6	0.6088 (4)	−0.02370 (11)	0.07416 (10)	0.0524 (6)	
O7W	0.7879 (4)	0.02001 (14)	0.54018 (13)	0.0592 (7)	
H7WA	0.849 (4)	0.0467 (13)	0.5203 (16)	0.071*	
H7WB	0.860 (5)	−0.0126 (12)	0.5454 (18)	0.071*	
O7	0.6937 (3)	−0.18124 (10)	0.15287 (10)	0.0434 (5)	
O8	0.8981 (3)	−0.10571 (11)	0.12730 (10)	0.0465 (6)	
O8W	1.1066 (5)	0.11321 (14)	0.68801 (14)	0.0824 (10)	
H8WB	1.097 (6)	0.1260 (18)	0.6478 (12)	0.099*	
H8WA	1.137 (6)	0.0734 (10)	0.6857 (19)	0.099*	
O9W	0.4790 (4)	0.40818 (12)	−0.04843 (13)	0.0514 (6)	
H9WA	0.552 (4)	0.3760 (13)	−0.0411 (14)	0.048 (11)*	
H9WB	0.401 (5)	0.3993 (19)	−0.0817 (15)	0.104 (19)*	
C1	0.6998 (4)	0.39104 (16)	0.79609 (15)	0.0417 (8)	
H1A	0.7074	0.3775	0.8404	0.050*	
C2	0.6795 (5)	0.45779 (17)	0.78211 (17)	0.0518 (9)	
H2A	0.6779	0.4881	0.8165	0.062*	
C3	0.6622 (5)	0.47783 (17)	0.71727 (19)	0.0530 (9)	
H3A	0.6446	0.5221	0.7068	0.064*	
C4	0.6708 (4)	0.43239 (16)	0.66644 (15)	0.0420 (8)	
C5	0.6501 (5)	0.44923 (19)	0.59653 (17)	0.0543 (10)	
H5A	0.6294	0.4927	0.5835	0.065*	
C6	0.6604 (5)	0.40298 (19)	0.54975 (16)	0.0521 (9)	
H6A	0.6444	0.4151	0.5048	0.062*	
C7	0.6953 (4)	0.33579 (17)	0.56737 (14)	0.0406 (8)	
C8	0.6990 (4)	0.36689 (15)	0.68516 (14)	0.0335 (7)	
C9	0.7180 (4)	0.31756 (15)	0.63583 (13)	0.0325 (7)	
C10	0.7060 (5)	0.28512 (19)	0.52134 (16)	0.0498 (9)	
H10A	0.6904	0.2943	0.4757	0.060*	
C11	0.7391 (5)	0.2229 (2)	0.54308 (15)	0.0512 (9)	
H11A	0.7459	0.1892	0.5125	0.061*	
C12	0.7631 (5)	0.20916 (17)	0.61220 (15)	0.0446 (8)	
H12A	0.7876	0.1662	0.6266	0.054*	
C13	0.6916 (4)	0.07233 (14)	0.22994 (13)	0.0324 (7)	
C14	0.7154 (4)	0.13290 (14)	0.20404 (13)	0.0326 (7)	
H14A	0.7379	0.1691	0.2323	0.039*	
C15	0.7059 (4)	0.14038 (14)	0.13487 (13)	0.0335 (7)	
C16	0.6709 (4)	0.08749 (14)	0.09286 (14)	0.0352 (7)	
H16A	0.6637	0.0925	0.0467	0.042*	
C17	0.6463 (4)	0.02584 (15)	0.12065 (14)	0.0375 (7)	
C18	0.6587 (4)	0.01780 (15)	0.18883 (14)	0.0373 (7)	
H18A	0.6453	−0.0235	0.2070	0.045*	
C19	0.7032 (4)	0.06265 (15)	0.30421 (13)	0.0364 (7)	
C20	0.7191 (4)	0.21662 (14)	0.04494 (12)	0.0303 (7)	
H20A	0.5913	0.2064	0.0225	0.036*	
H20B	0.8088	0.1896	0.0254	0.036*	
C21	0.7619 (4)	0.28832 (14)	0.03547 (13)	0.0301 (7)	
C22	0.5633 (5)	−0.08635 (15)	0.09867 (16)	0.0491 (9)	
H22A	0.4917	−0.1110	0.0620	0.059*	
H22B	0.4811	−0.0801	0.1323	0.059*	
C23	0.7349 (5)	−0.12653 (16)	0.12912 (13)	0.0366 (8)	

### Atomic displacement parameters (Å^2^)

**Table:**

	*U*^11^	*U*^22^	*U*^33^	*U*^12^	*U*^13^	*U*^23^
Co1	0.0424 (4)	0.0287 (3)	0.0329 (3)	0.0087 (3)	0.0163 (3)	0.0036 (2)
Co2	0.0366 (3)	0.0297 (3)	0.0316 (2)	0.00050 (19)	0.00464 (18)	−0.00106 (17)
N1	0.0316 (15)	0.0339 (15)	0.0350 (13)	0.0041 (12)	0.0014 (11)	−0.0024 (11)
N2	0.0332 (16)	0.0364 (16)	0.0336 (13)	−0.0016 (12)	0.0057 (11)	−0.0043 (11)
O1W	0.0514 (16)	0.0808 (19)	0.0353 (12)	−0.0249 (14)	0.0131 (12)	−0.0124 (12)
O1	0.106 (2)	0.0308 (14)	0.0483 (12)	0.0189 (13)	0.0372 (14)	0.0146 (10)
O2W	0.0503 (16)	0.0350 (13)	0.0458 (12)	0.0040 (12)	0.0035 (11)	−0.0021 (11)
O2	0.0557 (15)	0.0302 (12)	0.0309 (10)	0.0071 (10)	0.0126 (10)	0.0033 (9)
O3W	0.0424 (15)	0.0666 (17)	0.0352 (11)	−0.0129 (13)	0.0014 (11)	0.0045 (12)
O3	0.0793 (18)	0.0323 (13)	0.0291 (10)	−0.0105 (12)	0.0054 (11)	0.0057 (9)
O4	0.0784 (17)	0.0317 (13)	0.0357 (11)	−0.0141 (12)	0.0151 (11)	−0.0048 (10)
O4W	0.0341 (14)	0.0287 (13)	0.0752 (16)	−0.0024 (11)	−0.0048 (12)	0.0041 (12)
O5W	0.120 (2)	0.0343 (14)	0.0587 (14)	0.0220 (15)	0.0592 (15)	0.0090 (12)
O5	0.0533 (14)	0.0325 (12)	0.0288 (10)	−0.0056 (10)	0.0066 (10)	0.0048 (9)
O6W	0.0620 (17)	0.0366 (14)	0.0452 (12)	0.0151 (12)	0.0291 (12)	0.0126 (10)
O6	0.0850 (19)	0.0275 (13)	0.0391 (11)	0.0028 (12)	−0.0075 (12)	−0.0026 (10)
O7W	0.0489 (17)	0.0568 (18)	0.0733 (16)	0.0085 (13)	0.0140 (13)	0.0192 (14)
O7	0.0420 (14)	0.0323 (13)	0.0552 (13)	0.0002 (11)	0.0058 (11)	0.0082 (10)
O8	0.0469 (16)	0.0452 (15)	0.0470 (13)	−0.0055 (12)	0.0065 (11)	0.0075 (10)
O8W	0.115 (3)	0.0616 (19)	0.0827 (19)	0.0341 (19)	0.0517 (19)	0.0138 (16)
O9W	0.0445 (16)	0.0475 (17)	0.0584 (16)	0.0085 (14)	−0.0037 (13)	−0.0078 (13)
C1	0.040 (2)	0.041 (2)	0.0430 (17)	0.0031 (16)	0.0013 (15)	−0.0073 (15)
C2	0.051 (2)	0.042 (2)	0.061 (2)	0.0047 (18)	0.0050 (18)	−0.0142 (17)
C3	0.047 (2)	0.032 (2)	0.080 (3)	0.0069 (17)	0.0096 (19)	0.0036 (18)
C4	0.034 (2)	0.037 (2)	0.0546 (19)	0.0015 (16)	0.0040 (15)	0.0088 (16)
C5	0.046 (2)	0.049 (2)	0.067 (2)	0.0035 (19)	0.0087 (18)	0.0227 (19)
C6	0.046 (2)	0.062 (3)	0.048 (2)	0.0006 (19)	0.0068 (17)	0.0248 (18)
C7	0.0276 (18)	0.057 (2)	0.0373 (16)	−0.0055 (16)	0.0056 (14)	0.0055 (15)
C8	0.0247 (17)	0.0343 (18)	0.0404 (16)	−0.0015 (14)	0.0018 (13)	0.0021 (14)
C9	0.0231 (17)	0.0380 (19)	0.0360 (15)	−0.0038 (14)	0.0036 (13)	0.0057 (13)
C10	0.043 (2)	0.072 (3)	0.0354 (17)	−0.008 (2)	0.0093 (16)	0.0033 (18)
C11	0.046 (2)	0.070 (3)	0.0389 (18)	−0.006 (2)	0.0102 (16)	−0.0157 (17)
C12	0.045 (2)	0.044 (2)	0.0459 (18)	0.0017 (17)	0.0081 (16)	−0.0065 (16)
C13	0.0323 (18)	0.0343 (18)	0.0321 (15)	0.0024 (14)	0.0101 (13)	0.0015 (13)
C14	0.0398 (19)	0.0258 (17)	0.0329 (15)	0.0004 (14)	0.0081 (14)	0.0028 (12)
C15	0.0407 (19)	0.0265 (17)	0.0334 (15)	−0.0006 (14)	0.0059 (14)	0.0087 (13)
C16	0.040 (2)	0.0321 (18)	0.0327 (15)	0.0024 (15)	0.0025 (14)	0.0057 (13)
C17	0.042 (2)	0.0303 (18)	0.0378 (16)	0.0029 (15)	−0.0010 (14)	−0.0046 (13)
C18	0.044 (2)	0.0290 (17)	0.0388 (16)	0.0024 (15)	0.0048 (14)	0.0067 (13)
C19	0.041 (2)	0.0363 (19)	0.0353 (15)	0.0046 (16)	0.0155 (14)	0.0081 (14)
C20	0.0342 (18)	0.0302 (17)	0.0258 (14)	−0.0016 (14)	0.0025 (13)	0.0018 (12)
C21	0.0338 (18)	0.0262 (17)	0.0314 (15)	−0.0027 (14)	0.0086 (13)	0.0016 (13)
C22	0.061 (3)	0.0277 (19)	0.0520 (19)	−0.0018 (17)	−0.0109 (17)	−0.0066 (15)
C23	0.047 (2)	0.035 (2)	0.0256 (14)	0.0019 (17)	0.0007 (14)	−0.0030 (13)

### Geometric parameters (Å, °)

**Table:**

Co1—O5W^i^	2.0144 (19)	O8W—H8WB	0.845 (18)
Co1—O5W	2.0144 (19)	O8W—H8WA	0.842 (18)
Co1—O6W^i^	2.112 (2)	O9W—H9WA	0.833 (17)
Co1—O6W	2.112 (2)	O9W—H9WB	0.819 (18)
Co1—O7W	2.115 (3)	C1—C2	1.392 (5)
Co1—O7W^i^	2.115 (3)	C1—H1A	0.9300
Co2—O2W	2.072 (2)	C2—C3	1.358 (5)
Co2—O1W	2.093 (2)	C2—H2A	0.9300
Co2—O4W	2.102 (2)	C3—C4	1.390 (4)
Co2—N1	2.109 (2)	C3—H3A	0.9300
Co2—O3W	2.118 (2)	C4—C8	1.393 (4)
Co2—N2	2.157 (2)	C4—C5	1.437 (4)
N1—C1	1.321 (4)	C5—C6	1.344 (5)
N1—C8	1.362 (3)	C5—H5A	0.9300
N2—C12	1.327 (4)	C6—C7	1.426 (5)
N2—C9	1.348 (4)	C6—H6A	0.9300
O1W—H1WA	0.816 (17)	C7—C10	1.399 (5)
O1W—H1WB	0.839 (17)	C7—C9	1.415 (4)
O1—C19	1.244 (3)	C8—C9	1.436 (4)
O2W—H2WA	0.856 (17)	C10—C11	1.350 (5)
O2W—H2WB	0.846 (17)	C10—H10A	0.9300
O2—C19	1.266 (3)	C11—C12	1.406 (4)
O3W—H3WA	0.831 (17)	C11—H11A	0.9300
O3W—H3WB	0.788 (17)	C12—H12A	0.9300
O3—C15	1.372 (3)	C13—C14	1.361 (4)
O3—C20	1.412 (3)	C13—C18	1.384 (4)
O4—C21	1.241 (3)	C13—C19	1.501 (3)
O4W—H4WA	0.841 (17)	C14—C15	1.395 (3)
O4W—H4WB	0.837 (17)	C14—H14A	0.9300
O5W—H5WA	0.837 (17)	C15—C16	1.370 (4)
O5W—H5WB	0.826 (18)	C16—C17	1.398 (4)
O5—C21	1.261 (3)	C16—H16A	0.9300
O6W—H6WA	0.788 (17)	C17—C18	1.375 (4)
O6W—H6WB	0.810 (17)	C18—H18A	0.9300
O6—C17	1.375 (3)	C20—C21	1.511 (4)
O6—C22	1.424 (4)	C20—H20A	0.9700
O7W—H7WA	0.836 (18)	C20—H20B	0.9700
O7W—H7WB	0.834 (18)	C22—C23	1.515 (4)
O7—C23	1.266 (4)	C22—H22A	0.9700
O8—C23	1.239 (4)	C22—H22B	0.9700
			
O5W^i^—Co1—O5W	180.00 (8)	C2—C3—H3A	119.9
O5W^i^—Co1—O6W^i^	88.01 (8)	C4—C3—H3A	119.9
O5W—Co1—O6W^i^	91.99 (8)	C3—C4—C8	117.3 (3)
O5W^i^—Co1—O6W	91.99 (8)	C3—C4—C5	123.8 (3)
O5W—Co1—O6W	88.01 (8)	C8—C4—C5	118.9 (3)
O6W^i^—Co1—O6W	180.000 (1)	C6—C5—C4	120.9 (3)
O5W^i^—Co1—O7W	90.96 (12)	C6—C5—H5A	119.5
O5W—Co1—O7W	89.04 (12)	C4—C5—H5A	119.5
O6W^i^—Co1—O7W	91.08 (10)	C5—C6—C7	121.6 (3)
O6W—Co1—O7W	88.92 (10)	C5—C6—H6A	119.2
O5W^i^—Co1—O7W^i^	89.04 (12)	C7—C6—H6A	119.2
O5W—Co1—O7W^i^	90.96 (12)	C10—C7—C9	116.4 (3)
O6W^i^—Co1—O7W^i^	88.92 (10)	C10—C7—C6	124.6 (3)
O6W—Co1—O7W^i^	91.08 (10)	C9—C7—C6	118.9 (3)
O7W—Co1—O7W^i^	180.0	N1—C8—C4	123.0 (3)
O2W—Co2—O1W	91.48 (10)	N1—C8—C9	116.5 (3)
O2W—Co2—O4W	85.09 (9)	C4—C8—C9	120.5 (3)
O1W—Co2—O4W	86.82 (10)	N2—C9—C7	123.5 (3)
O2W—Co2—N1	165.12 (9)	N2—C9—C8	117.5 (2)
O1W—Co2—N1	99.13 (10)	C7—C9—C8	119.0 (3)
O4W—Co2—N1	85.11 (9)	C11—C10—C7	120.1 (3)
O2W—Co2—O3W	93.59 (10)	C11—C10—H10A	119.9
O1W—Co2—O3W	83.29 (9)	C7—C10—H10A	119.9
O4W—Co2—O3W	169.99 (9)	C10—C11—C12	119.8 (3)
N1—Co2—O3W	97.97 (10)	C10—C11—H11A	120.1
O2W—Co2—N2	95.03 (9)	C12—C11—H11A	120.1
O1W—Co2—N2	164.28 (10)	N2—C12—C11	122.2 (3)
O4W—Co2—N2	107.96 (10)	N2—C12—H12A	118.9
N1—Co2—N2	77.42 (9)	C11—C12—H12A	118.9
O3W—Co2—N2	82.04 (8)	C14—C13—C18	121.1 (2)
C1—N1—C8	117.2 (3)	C14—C13—C19	120.8 (3)
C1—N1—Co2	127.8 (2)	C18—C13—C19	118.1 (3)
C8—N1—Co2	114.98 (19)	C13—C14—C15	119.6 (3)
C12—N2—C9	118.0 (3)	C13—C14—H14A	120.2
C12—N2—Co2	128.2 (2)	C15—C14—H14A	120.2
C9—N2—Co2	113.33 (17)	C16—C15—O3	124.6 (2)
Co2—O1W—H1WA	131 (2)	C16—C15—C14	120.6 (3)
Co2—O1W—H1WB	117 (2)	O3—C15—C14	114.8 (3)
H1WA—O1W—H1WB	108 (2)	C15—C16—C17	118.7 (3)
Co2—O2W—H2WA	111 (2)	C15—C16—H16A	120.6
Co2—O2W—H2WB	118 (2)	C17—C16—H16A	120.6
H2WA—O2W—H2WB	99 (2)	C18—C17—O6	124.8 (3)
Co2—O3W—H3WA	122 (2)	C18—C17—C16	121.1 (3)
Co2—O3W—H3WB	127 (2)	O6—C17—C16	114.1 (2)
H3WA—O3W—H3WB	109 (3)	C17—C18—C13	118.9 (3)
C15—O3—C20	119.3 (2)	C17—C18—H18A	120.6
Co2—O4W—H4WA	126 (2)	C13—C18—H18A	120.6
Co2—O4W—H4WB	129 (2)	O1—C19—O2	123.0 (2)
H4WA—O4W—H4WB	103 (2)	O1—C19—C13	117.9 (3)
Co1—O5W—H5WA	121 (2)	O2—C19—C13	119.0 (3)
Co1—O5W—H5WB	128 (2)	O3—C20—C21	109.0 (2)
H5WA—O5W—H5WB	107 (2)	O3—C20—H20A	109.9
Co1—O6W—H6WA	128 (2)	C21—C20—H20A	109.9
Co1—O6W—H6WB	119 (2)	O3—C20—H20B	109.9
H6WA—O6W—H6WB	112 (3)	C21—C20—H20B	109.9
C17—O6—C22	116.8 (2)	H20A—C20—H20B	108.3
Co1—O7W—H7WA	119 (2)	O4—C21—O5	125.2 (3)
Co1—O7W—H7WB	115 (3)	O4—C21—C20	119.3 (2)
H7WA—O7W—H7WB	103 (2)	O5—C21—C20	115.5 (2)
H8WB—O8W—H8WA	103 (2)	O6—C22—C23	114.4 (3)
H9WA—O9W—H9WB	107 (3)	O6—C22—H22A	108.6
N1—C1—C2	123.5 (3)	C23—C22—H22A	108.6
N1—C1—H1A	118.3	O6—C22—H22B	108.6
C2—C1—H1A	118.3	C23—C22—H22B	108.6
C3—C2—C1	118.7 (3)	H22A—C22—H22B	107.6
C3—C2—H2A	120.7	O8—C23—O7	125.8 (3)
C1—C2—H2A	120.7	O8—C23—C22	119.9 (3)
C2—C3—C4	120.2 (3)	O7—C23—C22	114.3 (3)
			
O2W—Co2—N1—C1	−114.6 (4)	Co2—N2—C9—C8	5.0 (3)
O1W—Co2—N1—C1	20.4 (3)	C10—C7—C9—N2	1.2 (4)
O4W—Co2—N1—C1	−65.6 (3)	C6—C7—C9—N2	−179.7 (3)
O3W—Co2—N1—C1	104.8 (3)	C10—C7—C9—C8	−177.0 (3)
N2—Co2—N1—C1	−175.2 (3)	C6—C7—C9—C8	2.1 (4)
O2W—Co2—N1—C8	63.3 (4)	N1—C8—C9—N2	−2.8 (4)
O1W—Co2—N1—C8	−161.7 (2)	C4—C8—C9—N2	177.6 (3)
O4W—Co2—N1—C8	112.3 (2)	N1—C8—C9—C7	175.5 (3)
O3W—Co2—N1—C8	−77.3 (2)	C4—C8—C9—C7	−4.1 (4)
N2—Co2—N1—C8	2.66 (19)	C9—C7—C10—C11	−0.8 (5)
O2W—Co2—N2—C12	17.1 (3)	C6—C7—C10—C11	−179.8 (3)
O1W—Co2—N2—C12	−97.0 (4)	C7—C10—C11—C12	−0.2 (5)
O4W—Co2—N2—C12	103.6 (3)	C9—N2—C12—C11	−0.5 (5)
N1—Co2—N2—C12	−175.9 (3)	Co2—N2—C12—C11	170.9 (2)
O3W—Co2—N2—C12	−75.8 (3)	C10—C11—C12—N2	0.9 (5)
O2W—Co2—N2—C9	−171.1 (2)	C18—C13—C14—C15	0.2 (4)
O1W—Co2—N2—C9	74.8 (4)	C19—C13—C14—C15	179.3 (3)
O4W—Co2—N2—C9	−84.6 (2)	C20—O3—C15—C16	2.9 (4)
N1—Co2—N2—C9	−4.08 (19)	C20—O3—C15—C14	−177.2 (3)
O3W—Co2—N2—C9	96.0 (2)	C13—C14—C15—C16	0.7 (5)
C8—N1—C1—C2	0.5 (5)	C13—C14—C15—O3	−179.2 (3)
Co2—N1—C1—C2	178.4 (2)	O3—C15—C16—C17	179.5 (3)
N1—C1—C2—C3	2.1 (5)	C14—C15—C16—C17	−0.5 (5)
C1—C2—C3—C4	−2.0 (5)	C22—O6—C17—C18	5.8 (5)
C2—C3—C4—C8	−0.5 (5)	C22—O6—C17—C16	−173.9 (3)
C2—C3—C4—C5	178.8 (3)	C15—C16—C17—C18	−0.7 (5)
C3—C4—C5—C6	179.8 (3)	C15—C16—C17—O6	179.0 (3)
C8—C4—C5—C6	−0.9 (5)	O6—C17—C18—C13	−178.0 (3)
C4—C5—C6—C7	−1.1 (5)	C16—C17—C18—C13	1.6 (5)
C5—C6—C7—C10	179.4 (3)	C14—C13—C18—C17	−1.4 (5)
C5—C6—C7—C9	0.5 (5)	C19—C13—C18—C17	179.5 (3)
C1—N1—C8—C4	−3.2 (4)	C14—C13—C19—O1	−162.3 (3)
Co2—N1—C8—C4	178.6 (2)	C18—C13—C19—O1	16.8 (4)
C1—N1—C8—C9	177.2 (3)	C14—C13—C19—O2	17.1 (4)
Co2—N1—C8—C9	−1.0 (3)	C18—C13—C19—O2	−163.8 (3)
C3—C4—C8—N1	3.3 (5)	C15—O3—C20—C21	−178.3 (2)
C5—C4—C8—N1	−176.1 (3)	O3—C20—C21—O4	−0.9 (4)
C3—C4—C8—C9	−177.2 (3)	O3—C20—C21—O5	179.7 (2)
C5—C4—C8—C9	3.5 (4)	C17—O6—C22—C23	−81.3 (3)
C12—N2—C9—C7	−0.6 (4)	O6—C22—C23—O8	−4.5 (4)
Co2—N2—C9—C7	−173.3 (2)	O6—C22—C23—O7	177.7 (2)
C12—N2—C9—C8	177.7 (3)		

Symmetry codes: (i) −*x*+1, −*y*, −*z*+1.

### Hydrogen-bond geometry (Å, °)

**Table:**

*D*—H···*A*	*D*—H	H···*A*	*D*···*A*	*D*—H···*A*
O4W—H4WA···O7^ii^	0.84 (2)	1.88 (2)	2.713 (3)	173 (3)
O2W—H2WA···O8^ii^	0.86 (2)	1.86 (2)	2.690 (3)	163 (3)
O8W—H8WA···O1^ii^	0.84 (2)	1.91 (2)	2.732 (3)	163 (4)
O4W—H4WB···O2^iii^	0.84 (2)	1.91 (2)	2.731 (3)	169 (3)
O7W—H7WA···O9W^iii^	0.84 (2)	2.01 (2)	2.818 (3)	164 (3)
O8W—H8WB···O4^iii^	0.85 (2)	2.29 (3)	2.945 (3)	134 (4)
O3W—H3WA···O7^i^	0.83 (2)	2.12 (2)	2.881 (3)	152 (3)
O5W—H5WA···O1^i^	0.84 (2)	1.77 (2)	2.609 (3)	175 (4)
O1W—H1WB···O7^i^	0.84 (2)	1.88 (2)	2.711 (3)	170 (3)
O6W—H6WB···O5^iv^	0.81 (2)	2.00 (2)	2.812 (3)	177 (3)
O5W—H5WB···O4^iv^	0.83 (2)	1.89 (2)	2.715 (3)	174 (4)
O3W—H3WB···O4^iv^	0.79 (2)	2.25 (2)	3.029 (3)	168 (4)
O3W—H3WB···O3^iv^	0.79 (2)	2.54 (3)	3.080 (3)	127 (3)
O9W—H9WB···O2^v^	0.82 (2)	2.00 (2)	2.818 (3)	172 (4)
O7W—H7WB···O9W^vi^	0.83 (2)	1.97 (2)	2.805 (4)	174 (4)
O1W—H1WA···O5^vii^	0.82 (2)	1.97 (2)	2.785 (3)	172 (4)
O6W—H6WA···O2	0.79 (2)	2.00 (2)	2.775 (3)	169 (3)
O2W—H2WB···O8W	0.85 (2)	1.88 (2)	2.723 (3)	177 (3)
O9W—H9WA···O5	0.83 (2)	2.00 (2)	2.831 (3)	173 (3)

Symmetry codes: (ii) −*x*+2, −*y*, −*z*+1; (iii) *x*+1/2, −*y*+1/2, *z*+1/2; (i) −*x*+1, −*y*, −*z*+1; (iv) *x*−1/2, −*y*+1/2, *z*+1/2; (v) *x*−1/2, −*y*+1/2, *z*−1/2; (vi) −*x*+3/2, *y*−1/2, −*z*+1/2; (vii) *x*, *y*, *z*+1.
